# Transforming medicine: artificial intelligence integration in the peripheral nervous system

**DOI:** 10.3389/fneur.2024.1332048

**Published:** 2024-02-14

**Authors:** Yue Qian, Ahmad Alhaskawi, Yanzhao Dong, Juemin Ni, Sahar Abdalbary, Hui Lu

**Affiliations:** ^1^Rehabilitation Center, Hangzhou Wuyunshan Hospital (Hangzhou Institute of Health Promotion), Hangzhou, China; ^2^Department of Orthopedics, The First Affiliated Hospital, Zhejiang University, Hangzhou, China; ^3^Department of Orthopedic Physical Therapy, Faculty of Physical Therapy, Nahda University in Beni Suef, Beni Suef, Egypt; ^4^Alibaba-Zhejiang University Joint Research Center of Future Digital Healthcare, Zhejiang University, Hangzhou, China

**Keywords:** artificial intelligence, peripheral nervous system, neuro-prosthetic, pain management, neural network

## Abstract

In recent years, artificial intelligence (AI) has undergone remarkable advancements, exerting a significant influence across a multitude of fields. One area that has particularly garnered attention and witnessed substantial progress is its integration into the realm of the nervous system. This article provides a comprehensive examination of AI’s applications within the peripheral nervous system, with a specific focus on AI-enhanced diagnostics for peripheral nervous system disorders, AI-driven pain management, advancements in neuroprosthetics, and the development of neural network models. By illuminating these facets, we unveil the burgeoning opportunities for revolutionary medical interventions and the enhancement of human capabilities, thus paving the way for a future in which AI becomes an integral component of our nervous system’s interface.

## Introduction

Artificial intelligence (AI) in the peripheral nervous system represents a synergy between computational technologies and the complexities of neural networks. It aims to decode the intricacies of neural circuitry and develop advanced therapies to treat neurological disorders and enhance human performance (1, 2). This article explores the integration of AI in the peripheral nervous system, focusing on applications and the potential implications for medical science and neuro-engineering. Therefore, fostering integration among these applications holds the potential to catalyze the emergence of novel ideas and technologies aimed at advancing the field of peripheral nervous system injury management.

## AI-enhanced PNS disorders diagnostics

**Figure 1:**
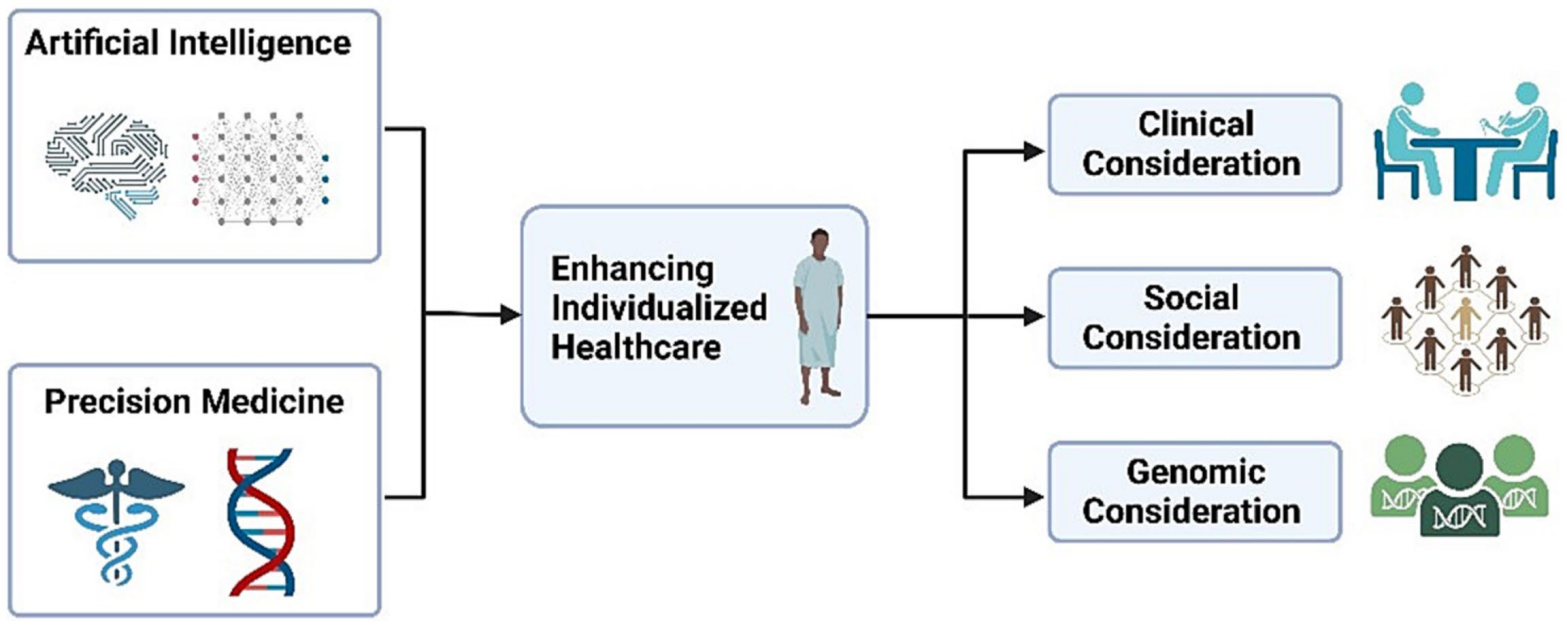
The integration of AI and precision medicine enhances individual healthcare by optimizing therapy planning and diagnostic methods.

Diagnosing PNS disorders is a complex and challenging task, requiring the integration of data from various sources and a comprehensive understanding of nerve function (3). AI, with its ability to process vast amounts of data and identify patterns that might be overlooked by human experts, offers tremendous potential to improve the accuracy and efficiency of PNS diagnostics (4–6). The PNS is composed of an intricate network of nerves that extend throughout the body. Disorders affecting the PNS can lead to a wide range of symptoms, including pain, weakness, numbness, and abnormal reflexes (7). Diagnosing these conditions involves a series of clinical assessments, electrophysiological tests, imaging studies, and sometimes invasive procedures such as nerve biopsies. However, the complex and often subtle nature of PNS disorders can make an accurate diagnosis a formidable challenge for healthcare professionals. Traditionally, PNS diagnostics have been heavily reliant on the expertise of neurologists and other specialists, which can lead to delays in diagnosis and potential misdiagnoses (8). Moreover, the interpretation of test results can be subjective and vary between different practitioners. A review indicated that in PNS-related cases, there was a delay in diagnosis in 82% of patients, largely due to misdiagnoses. This delay often resulted in nerve damage and disability, underscoring the critical nature of timely and accurate diagnosis in PNS disorders (9). In addition, the inherent variability and approximation in dermatome maps, which are used to diagnose PNS conditions, have been identified as factors contributing to delays in diagnosis and potential misdiagnoses. This variability can lead to confusion and inaccuracies in pinpointing neurological issues within the PNS (10). These limitations highlight the need for advanced technological solutions that can augment human expertise and improve the overall diagnostic process (Figure 1).

AI’s impact on PNS Image Analysis has been significantly transformed the field of neurology. The analysis of images from techniques like magnetic resonance imaging (MRI), computed tomography (CT), and electromyography (EMG) is critical for diagnosing various PNS disorders, including nerve injuries, neuropathies, tumors, and entrapment syndromes (11–14). Carpal tunnel syndrome (CTS) is caused by compression of the median nerve as it passes through the carpal tunnel in the wrist. AI algorithms can analyze EMG data and aid in identifying CTS with high sensitivity and specificity. Park et al. (15) utilized a machine learning-based methodology to explore the potential of assessing the severity of CTS by considering individual, clinical, and sonographic characteristics. The performance of all machine learning models surpassed a 70% accuracy threshold, with the extreme gradient boosting (XGB) model demonstrating the most impressive results. Moreover, the adoption of a one-versus-rest classification strategy further enhanced accuracy in contrast to the traditional multiclass classification approach.

Traditional manual analysis of these images is labor-intensive, time-consuming, and susceptible to human error. AI, particularly deep learning models, has shown tremendous potential in overcoming these limitations and revolutionizing PNS image analysis (16, 17).

Matsuda et al. (18) developed a method using deep learning models for analyzing images of soma and axons. This method is promising in predicting chemotherapy-induced peripheral neuropathy and understanding the mechanisms of action of different drugs. In the study by Umansky et al. (19), deep learning models were employed as a part of their innovative approach to analyze gait in mice following sciatic nerve injuries. Specifically, they utilized the Visual Gait Lab (VGL) deep learning system for gait analysis. This deep learning approach was used alongside standard manual gait and sensory assays as well as semi-automated analysis methods.

**Figure 2:**
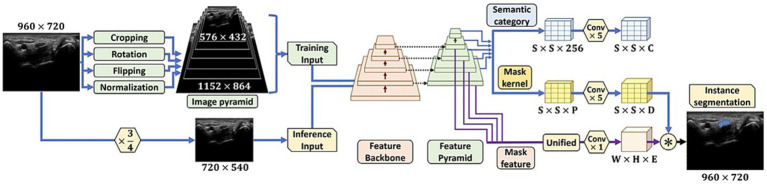
The initial steps for preparing images for analysis, and the layout of the SOLOv2-MN deep learning model. The figure initially shows how images are pre-processed, including normalization and augmentation techniques and provides visual representation of the SOLOv2-MN model’s architecture, critical for their method of automated median nerve segmentation in dynamic ultrasound imaging (24).

One of the primary challenges in PNS image analysis is the accurate segmentation of neural structures from background tissues. AI-based algorithms can automatically segment and label neural elements in MRI, CT, single photon emission computed tomography (SPECT) and positron emission tomography (PET) scans, allowing for precise anatomical localization (20–22). By identifying nerves, nerve roots, and other structures of interest, AI algorithms streamline the analysis process and provide neurologists with more comprehensive and detailed information for accurate diagnosis (11, 21). Chen et al. (23) explores the automation of quantifying axonal loss in patients with peripheral neuropathies. They developed a deep learning-derived muscle fat fraction (FF) method using a 3D U-Net computational model to segment muscle MRI images for individual muscle FF quantification. This approach significantly improved the efficiency and reduced the labor intensity of manual segmentations. Importantly, the study demonstrated good accuracy and agreement with manual methods. The findings suggest that this automated method can be valuable in the early detection of axonal loss in peripheral neuropathies. A study by Yeh et al. (24) focuses on the real-time automated segmentation of the median nerve in dynamic ultrasonography using deep learning. They developed a lightweight instance segmentation model, SOLOv2-MN, tailored for real-time segmentation of the median nerve in dynamic ultrasonography. This model outperformed several state-of-the-art models in terms of inference speed, while maintaining comparable segmentation accuracy. The study indicates that this model can be potentially integrated into the clinical setting to assist in the real-time diagnosis and evaluation of carpal tunnel syndrome using dynamic ultrasonography (Figure 2).

Other study explores the application of deep radiomics in diagnosing CTS by focusing on analyzing the deep radiomics features of median nerves using ultrasound images, which could potentially automate and improve the accuracy of CTS diagnosis (25).

**Figure 3:**
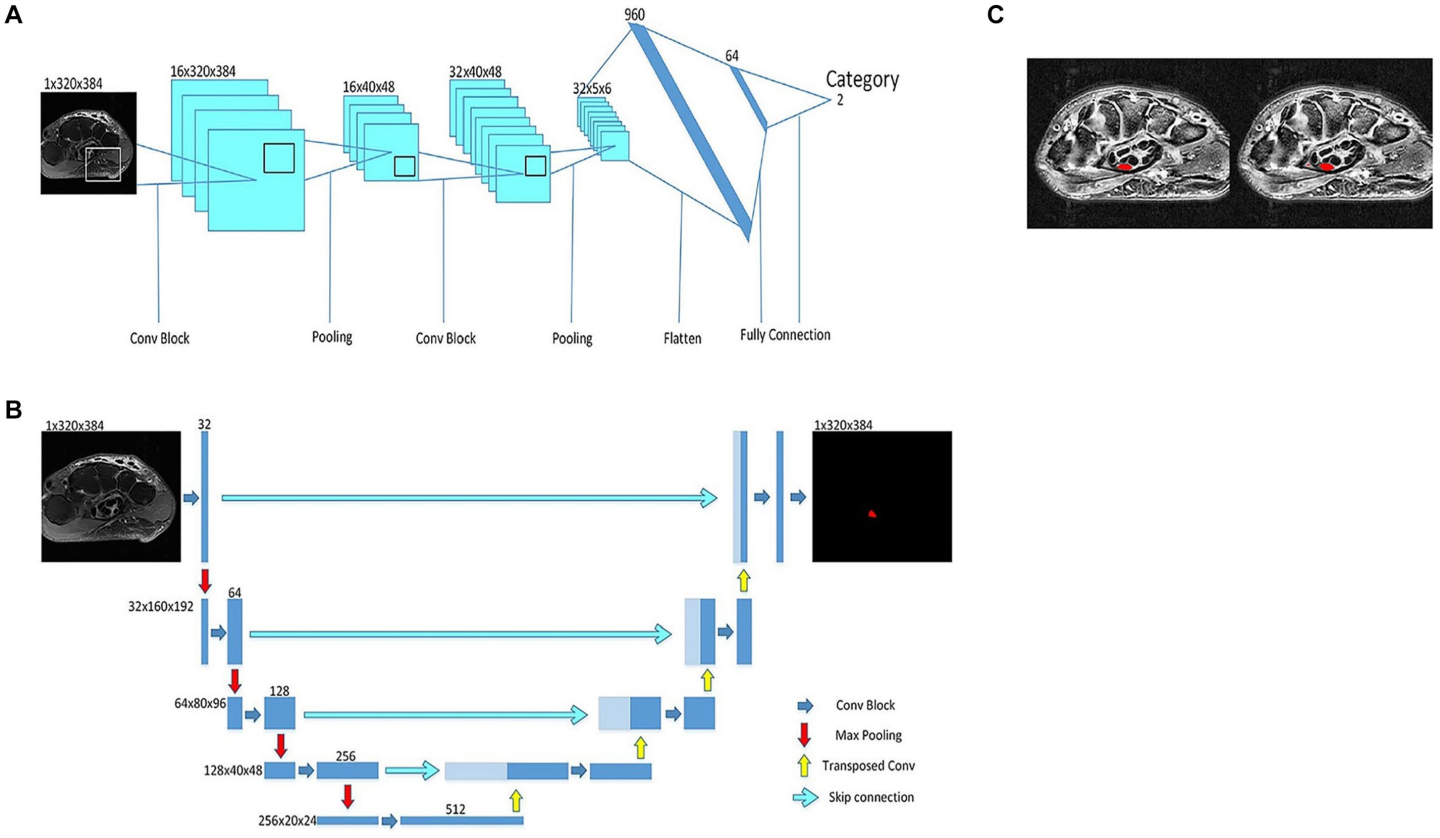
A deep neural network for MRI identification of carpal tunnel syndrome. **(A)** classification model to determine which MRI category. **(B)** U-Net for carpal tunnel area segmentation. **(C)** The result of the new proposed deep neural network, where the marker position (left) and predicted position (right) of a carpal tunnel.

AI models can be trained on large datasets of normal and abnormal images to learn subtle patterns and abnormalities in medical images that might go unnoticed by the human eye and assist in identifying and classifying PNS pathologies more accurately (21). This early detection facilitates timely interventions, potentially preventing further nerve damage and enhancing patient outcomes. Zhou et al. (26) present a deep learning framework called “Deep CTS” designed to address the challenges in segmenting the carpal tunnel region from MRI images, a critical aspect in the diagnosis and treatment of CTS. Deep CTS integrates a shape classifier with a simple convolutional neural network (Figure 3A) and a simplified U-Net for segmenting the carpal tunnel region (Figure 3B). This specialized structure is tailored specifically for the carpal tunnel, enabling efficient segmentation and improvement in the accuracy of intersection over union of results (Figure 3C). The article reports that this deep learning framework outperforms other segmentation networks for small objects. The model was trained with 333 images and tested with 82 images, achieving accuracy rate on reached 97.07% and a segmentation efficiency of 0.17 s. These results demonstrate the potential of Deep CTS for clinical application in accurately diagnosing CTS through MRI images. In addition, employing a cluster algorithm for the purpose of feature selection from datasets has yielded a notable degree of purity in the characterization of Guillain–Barré syndrome (GBS) through the application of AI. This outcome underscores a prospective pathway for the realization of computer-assisted GBS diagnosis (27). Furthermore, a study by Preston et al. (28) developed an AI algorithm using deep learning to classify peripheral neuropathy in diabetes and prediabetes. The algorithm achieved high precision, recall, and F1-score for both healthy participants and those with and without neuropathy. In the context of facial nerve paralysis, Song et al. (29) proposed a method for classifying facial nerve paralysis using a convolutional neural network (CNN) trained on clinical images, achieving 97.5% accuracy compared to neurologists’ assessments. While this study is specific to facial nerve paralysis, the approach might be adaptable to other peripheral nerve conditions. For epilepsy diagnosis, Krishnan et al. (30) evaluated DNNs using Gramian Angular Summation Field (GASF) images derived from EEG signals. A custom CNN showed high precision, recall, and F1-score in distinguishing between focal and normal GASF images, suggesting that DNNs could be a promising alternative for epilepsy detection, a condition often related to peripheral nervous system disorders. AI can fuse MRI and diffusion tensor imaging (DTI) data to map neural fiber trajectories and assess nerve integrity in cases of nerve injuries (31, 32). During certain procedures, such as nerve biopsies or nerve decompressions, real-time image analysis can be crucial for ensuring optimal surgical outcomes. DTI is based on the diffusion of water molecules in biological tissues. In peripheral nerves, the anisotropic diffusion of water can be quantified to assess nerve integrity. Key DTI metrics include fractional anisotropy (FA) and apparent diffusion coefficient (ADC), which provide information about nerve density, axonal diameter, and myelination. This technique is increasingly recognized as a valuable tool in peripheral imaging, particularly for assessing nerve abnormalities in conditions like diabetic peripheral neuropathy (DPN). DTI parameters, such FA and ADC, have been found effective in detecting nerve abnormalities in patients with type 2 diabetes and peripheral neuropathy. Decreased FA and increased ADC values are observed in the lumbosacral nerve roots of patients with DPN compared to healthy controls (33, 34). A study reported that acupoint injection of mecobalamin at Zusanli (ST36) could treat DPN and repair damaged nerves, as indicated by increased FA and decreased ADC in DTI (35). In addition, DTI provides reproducible measures of nerve microstructure and is used to assess the “health” of major nerves in the upper limb and its metrics vary with experimental conditions and the age of the subject (36). Furthermore, DTI has been used to evaluate the structure of lower limb muscles in chronic peripheral artery disease, which are not visible on traditional T1- and T2-weighted images (37).

## AI-based pain management

Pain management is a critical aspect of healthcare, particularly when dealing with conditions involving the peripheral nerve system. Neuropathic pain, caused by nerve damage or dysfunction, is a prevalent condition that can significantly impact a patient’s quality of life (38). Over the years, medical advancements, including the use of AI, have revolutionized pain management (39).

AI algorithms have demonstrated remarkable capabilities in pain assessment and diagnosis. These algorithms can analyze vast amounts of patient data, including medical history, imaging results, and sensory feedback, to identify patterns indicative of neuropathic pain (40). Matsangidou et al. (39) conducted a comprehensive systematic review delving into the clinical applications of machine learning (ML) in the context of pain. Their investigation unveiled compelling outcomes, particularly in the domain of pain intensity classification. The efficacy of ML techniques in this regard was substantiated across various medical conditions, encompassing sickle cell disease, spinal cord injury, osteoarthritis, evoked heat pain, low back pain, and thoracic pain, among others. Remarkably, their analysis showcased the versatility of machine learning algorithms in accurately categorizing pain intensity, irrespective of its underlying nature (39, 41–45). With the ability to recognize subtle features that may be overlooked by human clinicians, AI can aid in accurate and timely diagnosis, reducing the risk of misdiagnosis and providing targeted treatment options (40). The study by Coombes et al. (46) presents an eHealth intervention using Personal Activity Intelligence (PAI) for people with DPN. The intervention aimed to enhance physical activity self-management and examine its impact on foot symptoms. It involved weekly sessions with exercise tasters, behavior change counseling, and PAI self-monitoring. The results indicated significant reductions in aching and burning pain in the feet, suggesting the feasibility and potential benefits of the PAI eHealth intervention for managing pain in DPN patients.

Furthermore, AI-powered diagnostic tools can incorporate machine learning techniques, constantly improving their accuracy and efficiency. These tools can assist clinicians in selecting the most appropriate treatment plans tailored to each patient’s specific condition, leading to personalized and more effective pain management strategies (47). Amaya-Rodriguez et al. (48), showed that the use of machine learning is specifically employed in the context of identifying and analyzing druggable sites within the TRPV1 channel. This involves computational approaches that aid in drug discovery and repositioning for pain management. Machine learning algorithms play a pivotal role in understanding the molecular structure of the TRPV1 channel and its interaction with various compounds, thereby informing the development of targeted therapies in pain management. Multiple studies embarked on the creation of innovative models for pain recognition through the utilization of machine learning techniques. And each of these studies achieved remarkable success in accurately detecting instances of pain, showcasing commendable levels of accuracy in their outcomes (49–51). In a separate investigation, a cutting-edge deep-learning model was harnessed to automate pain assessment by analyzing facial expressions, a particularly valuable application in critically ill patients with a high accuracy rate (52). Magoon and Suresh (53) discusses the use of AI in various aspects of perioperative medicine, with a particular focus on pain management. They explore how AI can contribute to objective analgo-scoring and enhance the effectiveness of regional anesthesia. They specifically highlight the role of AI in improving the precision of ultrasound-guided nerve blocks, a critical component in pain management. AI’s predictive capabilities have proved instrumental in determining a patient’s response to different pain management interventions. Fleck et al. (54) examines neurocognitive predictors of adherence to an online pain self-management program for individuals undergoing long-term opioid therapy. The key findings indicate that selective attention and response inhibition/speed are significant predictors of program engagement. Furthermore, the study employs a machine learning approach to enhance the accuracy of predictions. These findings underscore the importance of cognitive factors in the effective management of chronic pain and adherence to self-management programs.

By analyzing historical patient data and treatment outcomes, AI algorithms can predict which interventions are more likely to be effective for specific neuropathic pain conditions. This predictive approach can significantly reduce the trial-and-error process, where patients may undergo various treatments before finding one that works for them (55–57). Moreover, AI can help identify potential side effects or complications associated with certain pain management medications or procedures, enabling clinicians to make informed decisions and minimize risks. This not only enhances patient safety but also leads to more cost-effective healthcare (57–59).

**Figure 4:**
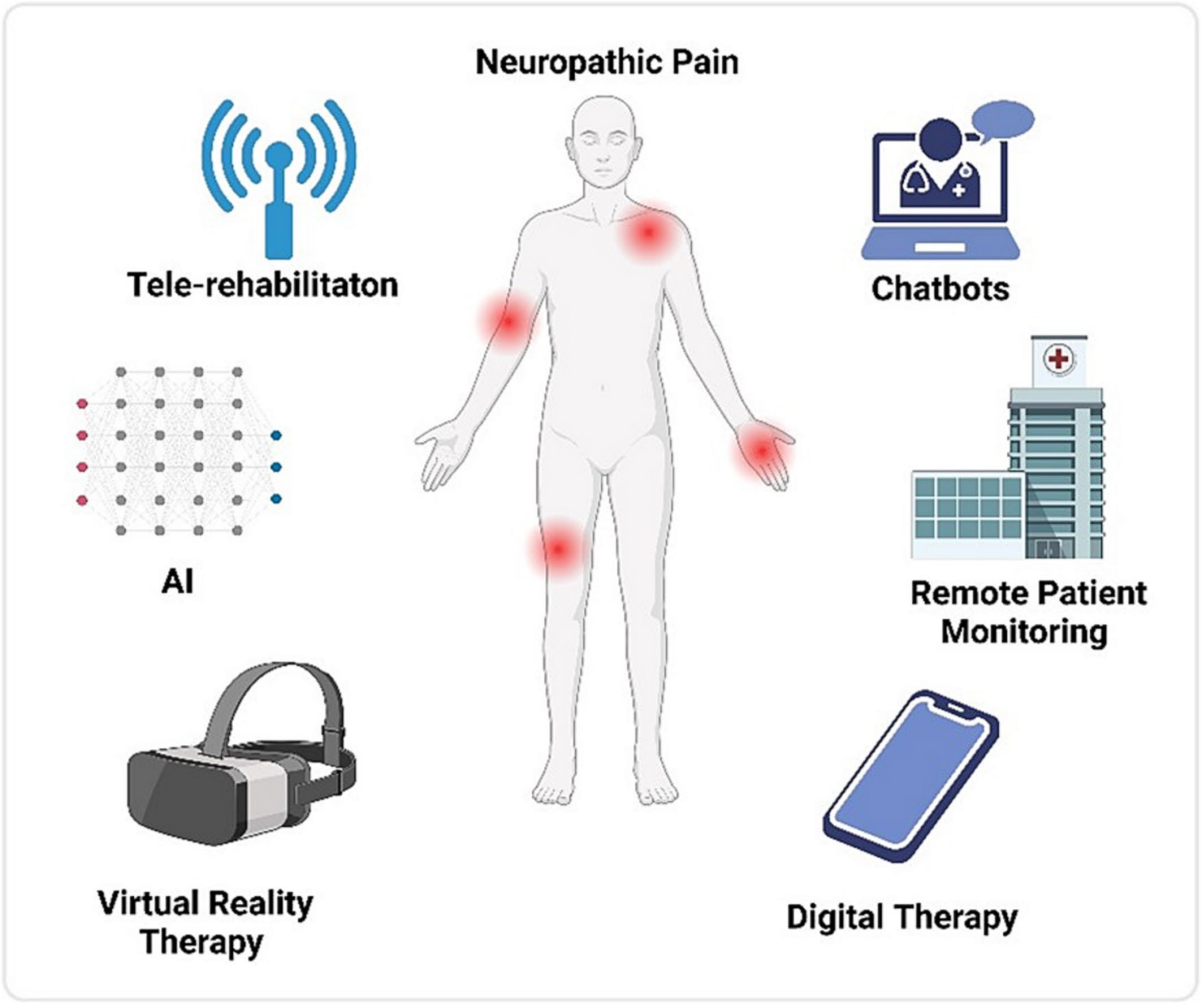
Different methods that help the patient to manage the neuropathic pain using artificial algorithms and neural networks.

AI plays a pivotal role in precision medicine, especially in the context of pain management. By analyzing genetic and molecular data, AI algorithms can identify specific biomarkers associated with certain neuropathic pain conditions. This information allows clinicians to predict a patient’s susceptibility to particular types of pain and helps design targeted therapies for optimal pain management outcomes (40, 60, 61). Zhao et al. (62) utilized AI in the form of the Weighted Gene Co-expression Network Analysis (WGCNA) algorithm. This method was applied to analyze RNA data, helping to identify modules and RNAs significantly associated with disease characterization in spinal cord injury (SCI) patients. The study also involved constructing co-expression networks and identifying pathways and biomarkers related to neuropathic pain post-SCI. Huang et al. (63) highlights the integration of advanced AI techniques in neuroimaging studies to understand and classify neuropathic pain conditions using specifically machine learning methods to analyze fMRI data of patients with herpes zoster (HZ) and postherpetic neuralgia (PHN). Their approach involved the use of support vector machine (SVM) algorithms for the classification of subjects based on brain activity patterns. The advent of AI in precision medicine opens up new possibilities for developing innovative treatments and breakthroughs in peripheral nerve system pain management. Pain management often involves non-pharmacological interventions to complement medical treatments. Virtual reality (VR) and distraction techniques have shown promise in reducing pain perception by diverting the patient’s attention away from the pain. Dy et al. (64) explored the usability and acceptability of VR for chronic pain management among a diverse group of patients in a safety-net healthcare setting. Using semi-structured interviews and observations of VR usage, the study found that most participants had no prior experience with VR for pain management but were interested in trying it. The results indicated that VR could be a usable and acceptable tool for chronic pain management, with many participants reporting positive experiences and expressing interest in future use. AI-driven VR experiences can be personalized based on a patient’s preferences and pain sensitivity levels (65, 66). Using AI to analyze patient feedback and physiological responses, VR systems can adapt in real-time to deliver content that provides the greatest distraction and pain relief. Additionally, AI can be used to monitor patient progress during VR therapy and adjust the treatment plan accordingly for optimal results (66–68) (Figure 4). Moreover, Suominen et al. (69) contributed to the advancement of pain assessment instruments. They proposed that pain-related textual notes could serve as fertile ground for the development of new pain assessment tools through the integration of human language technology, thereby promising a more refined and nuanced approach to pain evaluation.

## Neuro-prosthetics

AI and neuro-prosthetics are rapidly converging fields that hold immense promise in revolutionizing healthcare and improving the quality of life for individuals with neurological impairments (70). Neuro-prosthetics aims to restore lost sensory or motor functions by creating interfaces between the nervous system and external devices (71). Within this realm, advancements in AI have been instrumental in enhancing the efficacy and usability of neuro-prosthetic devices. In particular, AI’s integration into neuro-prosthetics in the PNS has opened up new possibilities for patients with amputations, paralysis, or sensory deficits (70).

For individuals who have suffered injuries or disorders affecting the PNS, controlling and interacting with their limbs becomes challenging, leading to motor impairments or sensory loss (72, 73). Neuro-prosthetics that target the PNS aims to bridge this gap by using external devices to interface with nerves, muscles, or sensory receptors, facilitating communication between the nervous system and the outside world (71).

One of the primary challenges in developing effective neuro-prosthetic devices lies in deciphering the complex neural signals generated by the PNS and translating them into meaningful actions for external prostheses or feedback for sensory restoration (71). This is where AI-powered signal processing techniques come into play. Machine learning algorithms, such as deep learning and neural networks, can process large datasets of neural signals to extract patterns and identify the intended movements or sensations (74).

For instance, AI algorithms have been used to decode neural signals from residual limb muscles in amputees, enabling intuitive and natural control of prosthetic limbs (75). By analyzing muscle activation patterns, AI can predict the intended movement and execute the corresponding action in the prosthetic limb. The study by Li et al. (76) presented a novel approach for planning and controlling a prosthetic arm using muscle-synergy-based methods and neural-adaptive control. The research focused on upper limb prosthetics, aiming to improve the motor control of artificial limbs through a better understanding of muscle synergies. They developed a muscle synergy-based framework that combines intention decoding with motion control, using surface electromyography (sEMG) signals for extracting muscle synergies. Additionally, they implemented a neural network-based control system for the prosthetic arm, demonstrating its effectiveness in practical applications with both healthy participants and an upper limb amputee. This approach has shown significant advancements in dexterity, allowing users to perform complex tasks with more ease and accuracy (77).

Apart from motor control, AI has been pivotal in providing sensory feedback to users of neuro-prosthetic devices. Sensory feedback is crucial for users to perceive the environment, exert the right amount of force, and interact safely. Sensory restoration in neuro-prosthetics involves recording sensory information from the PNS, transmitting it to the brain, and integrating it with other sensory inputs, often in real-time (78, 79). Ghildiyal et al. (80) applied electromyography pattern recognition for controlling prosthetic limbs using various machine learning techniques. They developed a force-controlled prosthetic limb that improves the self-reliance, quality of life, and mental strength of amputees. The study employed machine learning regression approaches, such as support vector regressor (SVR), linear regression, and random forest models, to predict the force required to regulate the voltage for the servomotors in the prosthetic limb. The Random Forest model provided the most accurate prediction for controlling the voltage and, consequently, the limb’s movements. AI algorithms play a central role in interpreting and integrating sensory data. They can recognize and classify different sensory stimuli, such as pressure, temperature, and texture, and relay this information back to the user through the neuro-prosthetic device. This feedback loop facilitates better control and enhances the sense of embodiment, making neuro-prosthetic devices feel like a natural extension of the user’s body (81). Hasse et al. (82) demonstrated the potential of machine learning-based functional electrical stimulation (FES) to evoke complex arm movements, they used FES combined with machine learning to control complex movements in paralyzed upper limbs. In addition, they recorded arm kinematics and electromyographic (EMG) activity from a “trainer” monkey making a range of arm movements. This data was used to train an artificial neural network (ANN) to predict muscle activity patterns. These patterns were converted into stimulus pulses delivered to muscles in paralyzed monkeys.

**Figure 5:**
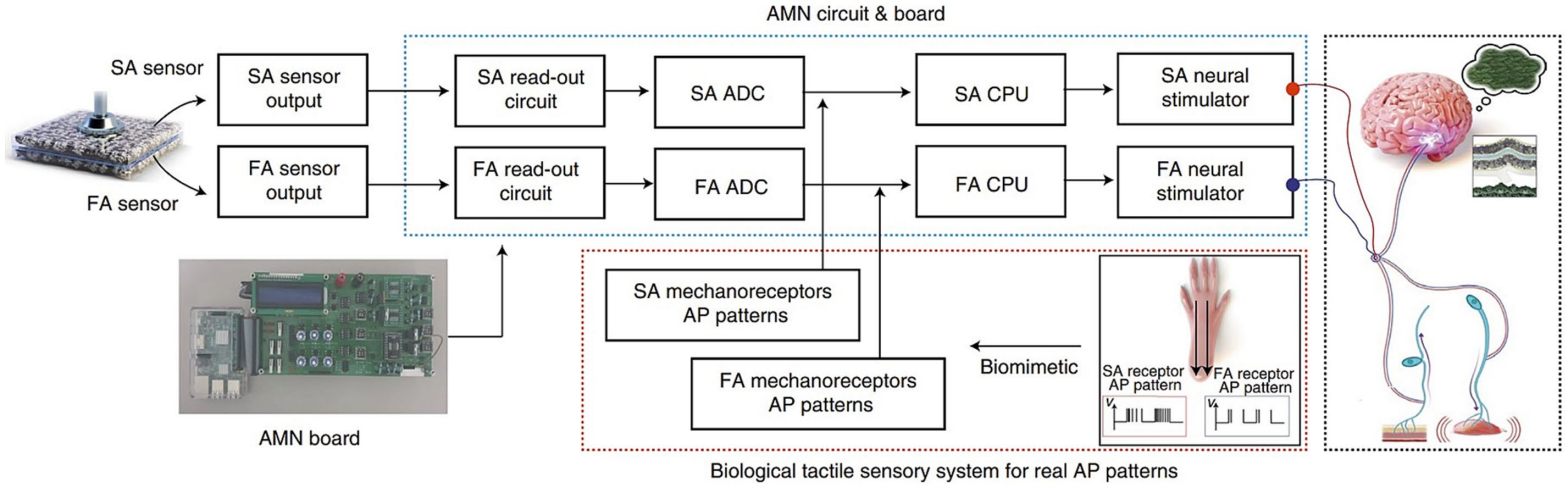
Illustrates the signal-processing procedure in an artificial tactile sensing system. It shows how signals from two types of sensors (SA and FA) are processed through an artificial mechanoreceptor neural board. This processing mimics the responses of real nerve cells to pressure and vibration stimuli, replicating the sensory functions of biological skin (81).

**Figure 6:**
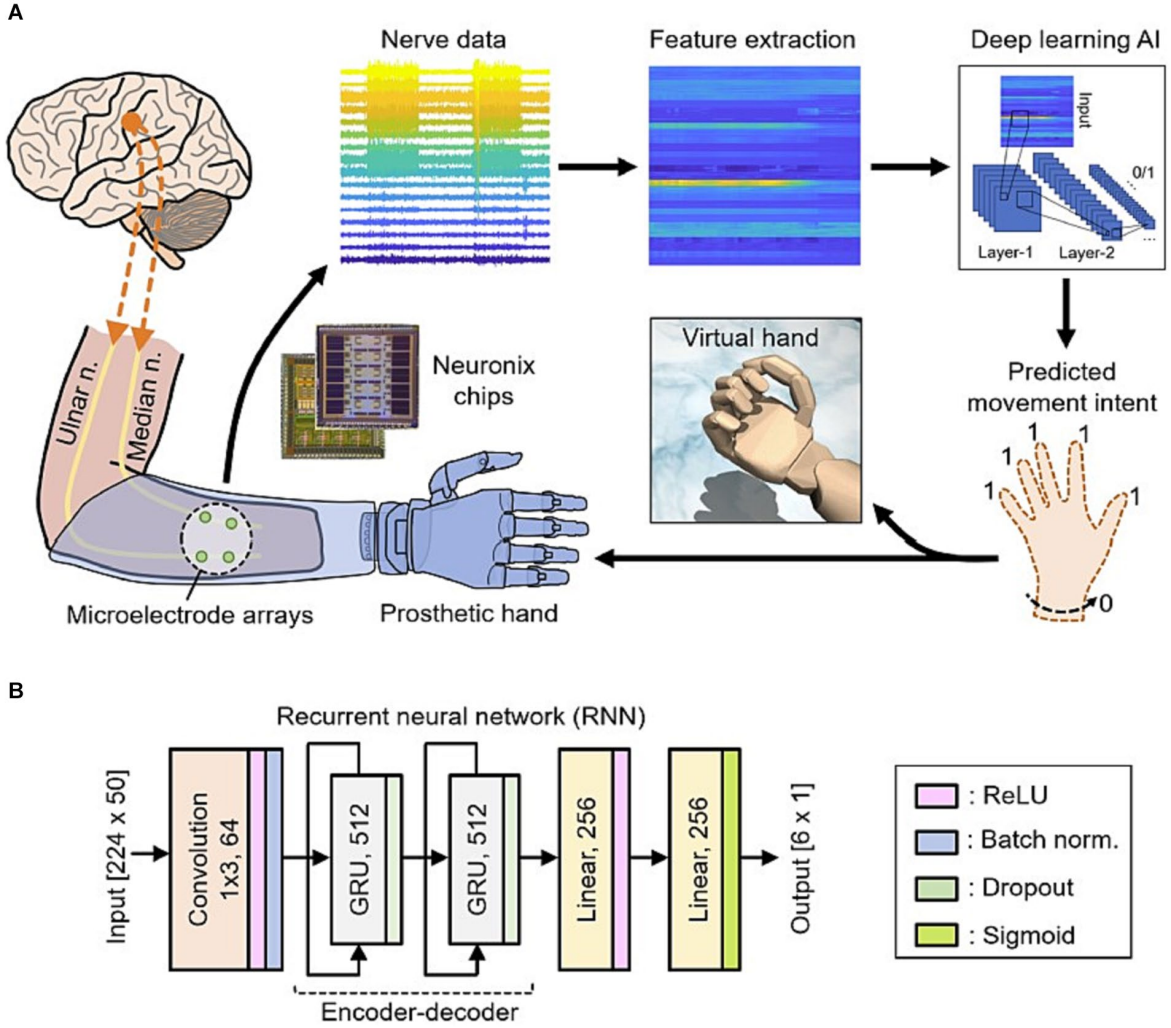
**(A)** Data from nerves in the individual’s amputated arm are collected using Neuronix neural interface chips. This is followed by the process of extracting key features. Subsequently, a deep learning AI utilizes these features to ascertain the individual’s intention to move multiple degrees of freedom at once. These predictions are then translated into real-time movements of either a virtual hand or a prosthetic hand. **(B)** The deep learning AI based on the recurrent neural network (RNN) architecture (88).

AI’s adaptability and capacity to learn from user interactions have enabled neuroprosthetic devices to evolve and personalize their functionality (71, 83). Traditional prostheses were often rigid and required manual adjustments to fit individual user needs (84, 85). However, AI-driven neuro-prosthetics can continuously adapt their control mechanisms based on the user’s behavior and preferences. For instance, AI algorithms can learn from a user’s neural signals to optimize control strategies, making the neuro-prosthetic more efficient over time. This adaptability allows users to fine-tune their control, resulting in a more natural and seamless experience with the device (86). Due to the constraints in material selection, sensory synaptic devices can only perform relatively simple functions and lack diverse synaptic characteristics. Consequently, a higher level of device structures must be devised to compensate for this limitation (87). Chun et al. (81) introduced an innovative artificial neural tactile skin system which emulates the intricate human tactile recognition mechanism through the utilization of particle-based polymer composite sensors along with a signal conversion setup. These sensors exhibit discerning responsiveness to pressure and vibration, mirroring the behavior of both slow adaptive and fast adaptive mechanoreceptors found in human skin (Figure 5). This intricate functionality enables the generation of output signal patterns reminiscent of sensory neurons. Moreover, they developed an artificial finger endowed with the capability to acquire the proficiency of categorizing intricate and multifaceted textures. This is achieved by seamlessly integrating the sensor signals with a cutting-edge deep learning technique (81). Luu et al. (88) have introduced a groundbreaking neuro-prosthetic system that showcases a fundamental principle by harnessing the capabilities of AI to translate an amputee’s intended movements via a peripheral nerve interface. Their study reveals that the AI neural decoder, implemented through this nerve interface, exhibits distinct characteristics of robust control over prosthetics. Through this AI agent, individuals with limb amputations can seamlessly command prosthetic upper limbs using their mental intentions, as the AI system deciphers their genuine motor intents (Figure 6). Furthermore, the AI agent demonstrates its potential in enabling intricate hand gestures, effectively decoding multiple degrees of freedom (DOF) simultaneously (88, 89). This aspect becomes particularly significant when coupled with substantial training data, allowing the agent to fully exploit the capabilities of near-anatomic prostheses. This advancement paves the way for diverse hand movements, fulfilling the essential prerequisites for achieving natural hand control. The efficacy of AI agent is substantiated by the hand matching task, underscoring its ability to deliver real-time performance with remarkable accuracy. Notably, the agent attains an impressive prediction accuracy exceeding 99% across all gestures, accompanied by minimal latency of approximately 0.81 s (88). This low latency translates to a substantial information throughput rate of 365.4 beats per minute (bpm), further emphasizing the agent’s efficacy and potential impact.

A pioneering study has conceptualized and demonstrated the inaugural utilization of graphdiyne-based artificial synapse components, boasting both parallel processing capabilities and adept information integration. This innovative approach involves the interconnection of the graphdiyne artificial synapse (GAS) with artificial motor neurons, resulting in the construction of synthetic efferent nerves, which, in turn, propel artificial muscles into motion (90). The GAS component exhibits the remarkable capacity to concurrently process multiple input sets, effectively amalgamating their respective outputs to exert precise control over the degree of artificial muscle flexion. This dynamic integration of parallel processing and output amalgamation holds significant promise for advanced control mechanisms (90, 91).

## Challenges and future directions

One significant challenge is the complexity of neural data interpretation. AI algorithms rely on vast datasets for training and validation, but neural signals can be highly variable and noisy (92). Developing AI models that accurately interpret and distinguish between different neural patterns is crucial for reliable diagnosis and treatment. Additionally, the adaptability of AI algorithms to individual variations in the PNS presents a formidable obstacle that demands sophisticated customization and optimization strategies. Data privacy and security also emerge as critical concerns (93). The transmission and storage of sensitive neural information for AI analysis raise ethical questions regarding patient consent, data ownership, and the potential for breaches (94, 95). Safeguarding patient privacy while harnessing the power of AI necessitates the establishment of robust security protocols and transparent regulatory frameworks. Furthermore, the successful integration of AI into the PNS requires interdisciplinary collaboration between medical professionals, neuroscientists, engineers, and computer scientists. Bridging the gap between these fields is essential to ensure seamless communication and effective translation of research findings into clinical applications (96). Transfer learning allows AI models trained on large datasets from one healthcare institution to be fine-tuned on smaller datasets from another institution, thus adapting the model to local patient demographics and variations in image acquisition protocols (39–41). Data augmentation techniques also help improve AI model performance by creating synthetic data variations, thereby increasing the diversity of training data and making the models more robust to unseen cases (42). The preeminence of research articles concentrated on the application of AI in the central nervous system within a prominent citation database highlights the pressing need for expanded research efforts dedicated to the peripheral nervous system. As for future directions, the potential of AI in the PNS is immense. Advances in neural interface technology and machine learning algorithms could lead to more precise and personalized treatments for neurological disorders. Enhanced real-time monitoring and adaptive interventions driven by AI could provide patients with proactive care and symptom management (97). AI algorithms can be deployed on the imaging systems used in the operating room to provide instant feedback to surgeons. By identifying critical structures and avoiding inadvertent damage (15, 98–100). Research efforts should also focus on the long-term effects of AI-PNS interactions. Safety, reliability, and the potential for neural plasticity need to be thoroughly investigated to understand the lasting impact of AI-based interventions on the nervous system (Table 1).

**Table 1:** Summary of AI application in the peripheral nervous system.

Application Area	AI utilization	Description
Neural signal processing	Signal enhancement	AI algorithms enhance weak neural signals by reducing noise and improving signal-to-noise ratio
	Feature extraction	AI identifies relevant features in neural signals for better understanding and analysis
	Pattern recognition	AI recognizes patterns in neural signals, aiding in the diagnosis of neurological disorders
	Signal decoding	AI decodes neural signals to control prosthetic devices, restoring motor function in patients
Neuroimaging analysis	Image enhancement	AI improves the quality of neuroimages, aiding in clearer visualization of peripheral structures
	Lesion detection	AI assists in identifying lesions or abnormalities in peripheral nerves from neuroimaging data
	Nerve segmentation	AI automates the process of segmenting peripheral nerves from imaging data for quantitative analysis
Diagnostics	Disease classification	AI algorithms classify peripheral nerve disorders based on symptoms, patient history, and data
	Risk prediction	AI helps assess the risk of developing peripheral nerve problems by analyzing various factors
	Early detection	AI aids in the early identification of peripheral nerve dysfunction, enabling timely interventions
Assistive devices	Prosthetic control	AI allows amputees to control prosthetic limbs through neural signals, restoring limb functionality
	Exoskeletons	AI-powered exoskeletons assist individuals with mobility impairments, adapting to their movements
	Sensory feedback	AI enables the integration of sensory feedback into prosthetic devices, enhancing user experience
Pain management	Predictive modeling	AI predicts pain levels based on neural responses, assisting in personalized pain management plans
	Treatment optimization	AI optimizes pain treatment regimens by analyzing patient responses and adjusting therapies
	Neurostimulation	AI-controlled neurostimulation devices deliver targeted therapy for pain relief in specific conditions
Research	Data analysis	AI analyzes large-scale neural datasets, revealing insights into peripheral nervous system functions
	Drug development	AI accelerates drug discovery by simulating neural interactions and predicting potential drug candidates
	Modeling and simulation	AI-based models simulate peripheral nerve behavior, aiding in understanding neural responses

## Conclusion

The integration of artificial intelligence (AI) into the peripheral nervous system (PNS) marks a remarkable advancement in the field of medical technology. The utilization of AI in the PNS holds immense promise for diagnosing, monitoring, and treating various neurological disorders, thereby enhancing the quality of life for countless individuals. Through the synergy of AI algorithms and neural interfaces, we are witnessing the development of innovative solutions that bridge the gap between the biological and technological realms. The incorporation of AI enables real-time data analysis, providing healthcare professionals with unprecedented insights into neural activities and patterns. This, in turn, leads to more accurate diagnoses and personalized treatment strategies tailored to each patient’s unique neural response. Moreover, the application of AI in the PNS holds the potential to restore lost sensory or motor functions, offering renewed independence to those with disabilities.

## Author contributions

YQ: Data curation, Writing – original draft. AA: Data curation, Writing – original draft. YD: Writing – review & editing. JN: Writing – review & editing. SA: Conceptualization, Investigation, Supervision, Writing – review & editing. HL: Conceptualization, Investigation, Supervision, Writing – review & editing.
